# Junction-free Flat Copper Nanofiber Network-based Transparent Heater with High Transparency, High Conductivity, and High Temperature

**DOI:** 10.1038/s41598-018-32045-6

**Published:** 2018-09-11

**Authors:** Geon Hwee Kim, Jung Hwal Shin, Taechang An, Geunbae Lim

**Affiliations:** 1 Department of Mechanical Engineering, Pohang University of Science and Technology (POSTECH), Pohang, 790-784 Republic of Korea; 2 School of Mechanical Engineering, Kyungnam University, 7 KyungnamdaehakCo, Masanhappo-gu, Changwon, Gyeongsangnam-do 51767 Republic of Korea; 3 Department of Mechanical Design Engineering, Andong National University, Kyungbuk, 760-749, Andong, Republic of Korea

## Abstract

Transparent conducting electrodes (TCE) are widely used in a variety of applications including displays, light-emitting diodes (LEDS), and solar cells. An important factor in TCE design is active control of the sheet resistance and transparency; as these are inversely proportional, it is essential to develop a technology that can maintain high transparency, while actively controlling sheet resistance, for a range of applications. Here, a nanofiber network was fabricated based on direct electrospinning onto a three-dimensional (3-D) complex substrate; flat metal electrodes without junction resistance were produced using heat treatment and electroless deposition. The fabricated transparent electrode exhibited a transparency of over 90% over the entire visible light range and a sheet resistance of 4.9 ohms/sq. Adhesion between the electrode and substrate was superior to other electrospinning-based transparent electrodes. The performance of the transparent electrode was verified by measurements taken while using the electrode as a heater; a maximum temperature of 210 °C was achieved. The proposed copper nanofiber-based heater electrode offers the advantages of transparency as well as application to complex 3-D surfaces.

## Introduction

Transparent conducting electrodes are used in displays^1^, light-emitting diodes (LEDs)^2^, touchscreens^3^, solar cells^4^, and other technologies. Indium tin oxide (ITO), the most commonly used material for TCEs, is expensive and brittle, limiting its application. A number of studies have been conducted on TCEs made of carbon nanotubes (CNTs)^5^, graphene^6^, metal nanowires^7^, and conductive polymers^8^ to replace ITO. Most applications require high transparency and low sheet resistance. One-dimensional (1-D) metal nanowire networks have the advantage of having relatively low sheet resistance for the same transparency^9^. However, to improve performance, it is necessary to reduce the contact resistance caused by the intersection of the nanowire. Therefore, a number of studies using mechanical pressing^10^ or thermal post-processing^11^ have been conducted to reduce the contact resistance; however, these processes are complicated and reduce the choice of substrate.

Long nanowires have been fabricated in an attempt to overcome the limitations of 1-D metal nanowire networks; additionally, numerous studies have been carried out to reduce the number of junctions, which is the biggest contributor to the increase in sheet resistance^12^. Electrospinning is one of the most popular techniques for fabricating ultra-thin nanofibers economically, with features such as extremely long lengths, a high surface area-to-volume ratio, a small pore size, and high porosity. These advantages of electrospinning are used to fabricate gas sensors^13^, filters^14^, batteries^15^ and fabrics^16^. Recently, the electrospinning technique for making transparent electrodes has been developed. An *et al*.^9^ collected electrospun polymer fibers and metallized these by copper electroplating for transfer to the desired substrate for transparent electrode fabrication. Jo *et al*.^17^ demonstrated a heater application with electrodes fabricated using a similar technique^9^. Wu *et al*.^18^ fabricated a transparent electrode by electrospinning an electrode template then fabricating a metal nanotrough using low step coverage via a metal deposition process; the prepared electrode was transferred to the desired location to form a surface electrode. Hsu *et al*.^19^ demonstrated electrospinning of a nanofiber embedded with SnCl_2_ directly onto a hydrophobic coated substrate, which was then metallized by electroless deposition. Azuma *et al*.^20^ used an electrospun nanofiber network as a masking layer for aluminum wet etching and fabricated a transparent electrode with this metal network.

However, these techniques are characterized by the fact that the electrodes are made in a floating state and transferred to the target substrate or have a core layer of polymer. The transfer process has an adhesion problem at the interface between the substrate and the electrode and requires precise transfer conditions to uniformly form the electrodes on the surface. The complexity of this transfer process results in limitations in substrate shape selection and large-area fabrication. If the polymer core layer remains after the process, the electrospun nanofiber network will significantly reduce the conductivity; further disadvantages arise from the waste of materials and the use of metal etchant.

In this study, we fabricated a random network copper electrode with a flat two-dimensional (2-D) shape by electrospinning directly onto a complex three-dimensional (3-D) substrate. The metal network part of the fabricated transparent electrode has resistance-free junctions and 2-D morphology. This feature improves the electrical characteristics of the transparent electrode and ensures mechanical durability. Given that the seed layer to be subjected to electroless deposition is formed through a heat treatment process, the polymer component generated in the electrospinning process disappears. Because a full solution process is used with no vacuum process or electrodeposition, the method can be used for large-scale production. In addition, the sheet resistance and transparency can be controlled easily by adjusting the deposition time; this control is necessary because different sheet resistances are required depending on the application^21^. The performance of the transparent electrode was verified by measurements taken while using the electrode as a heater; the heater was robust and able to reach temperatures above 200 °C.

## Experimental Section

### Materials

Polyvinylpyrrolidone (PVP, AR, M.W. 1,300,000) powder was purchased from Alfa Aesar. Formaldehyde solution was purchased from Wako Pure Chemical Industries. Ammonium tetrachloropalladate ((NH_4_)_2_PdCl_4_) was purchased from Dongjin PGMs Chemical Company. Sodium hydroxide (NaOH) and potassium sodium (+) – tartrate tetrahydrate was purchased from Kanto Chemical Co., Inc. N,N-dimethylmethanamide AR (DMF) was purchased from Sigma Aldrich, and copper (II) sulfate pentahydrate was purchased from Daejung Chemicals & Metals Co., Ltd. All reagents were used as received and without further purification.

### Seed layer fabrication

**Figure 1:**
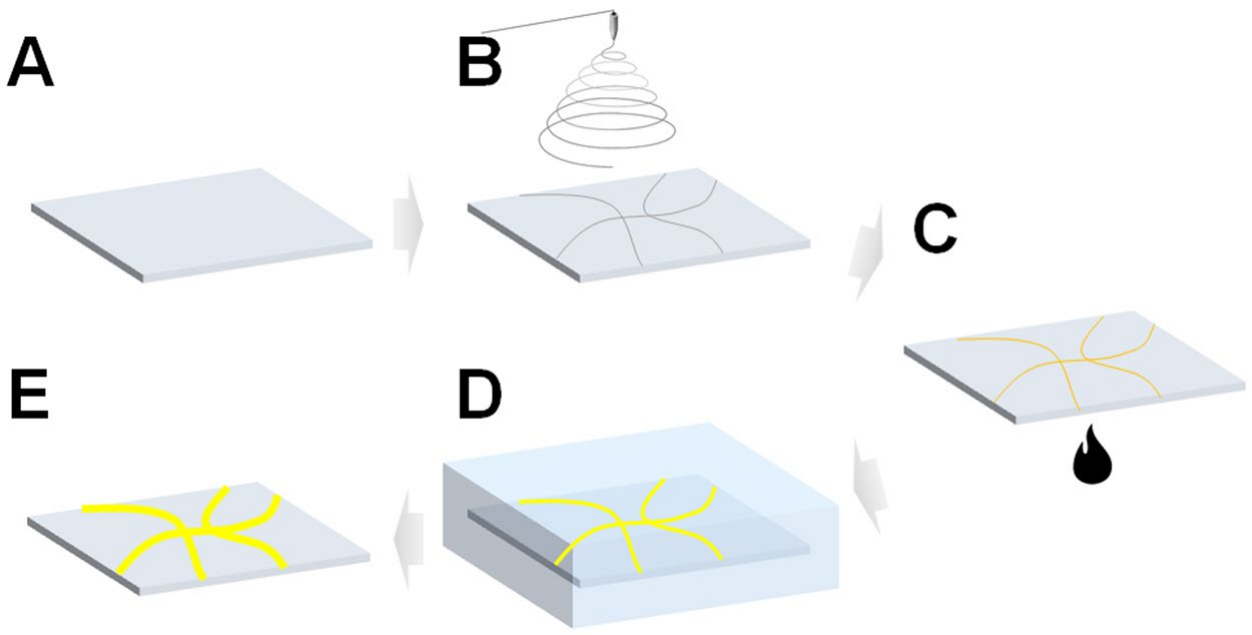
Schematic illustration of the transparent electrode fabrication process. (**A**) Preparation of bare glass substrate. (**B**) Electrospinning of palladium-embedded nanofibers. (**C**) Calcination to eliminate the polymer component of the nanofiber. (**D**) Copper electroless deposition. (**E**) Fabricated transparent electrode.

First, the substrate and a polymer solution containing palladium ions were prepared for electrospinning (Fig. 1A). The polymer solution was prepared by adding 0.1 g/mL PVP and 0.03 g/mL (NH_4_)_2_PdCl_4_ to DMF and stirring at 600 rpm with a magnetic stirrer for 2 h. The electrospinning conditions were as follows: a distance of 10 cm between the tip and collector, an applied voltage of 15 kV, and a solution flow rate of 0.1 mL/min. A motorized biaxial stage was set up to control the amount of nanofiber collected on the substrate (Fig. 1B). Once the nanofiber was collected on the glass substrate, the polymer was decomposed and calcined in air at 500 °C to form a seed layer for electroless deposition (Fig. 1C).

### Copper electroless deposition

Copper electroless deposition was performed by adding 0.1 mL/mL, 40 mg/mL, 140 mg/mL, and 30 mg/mL of formaldehyde, sodium hydroxide, potassium sodium (+) - tartrate tetrahydrate and copper(II) sulfate pentahydrate in DI water. The degree of electrode formation was adjusted by controlling the reaction time of the substrate on which the seed layer was formed (Fig. 1D,E).

### Characterization

The morphology of the copper nanostructure was determined using a scanning electron microscope equipped for energy dispersive X-ray spectroscopy (EDS) (TESCAN LYRA 3 XMH). The width was determined using Image J software. The morphologies and thicknesses of the nanostructures were determined using atomic force microscopy (AFM, Digital Instruments Multimode Nanoscope III) in tapping mode. The transmittance and sheet resistance of the copper nanostructure were characterized by ultraviolet-visible (UV-Vis) spectrophotometry (Agilent 8453, set the transmittance of the glass substrate to the reference transmittance) and the four-point probe technique using Keithley 2182 A and 6221 units, respectively. Infrared (IR) images of the copper electrodes were measured using Testo 868.

## Results and Discussion

**Figure 2:**
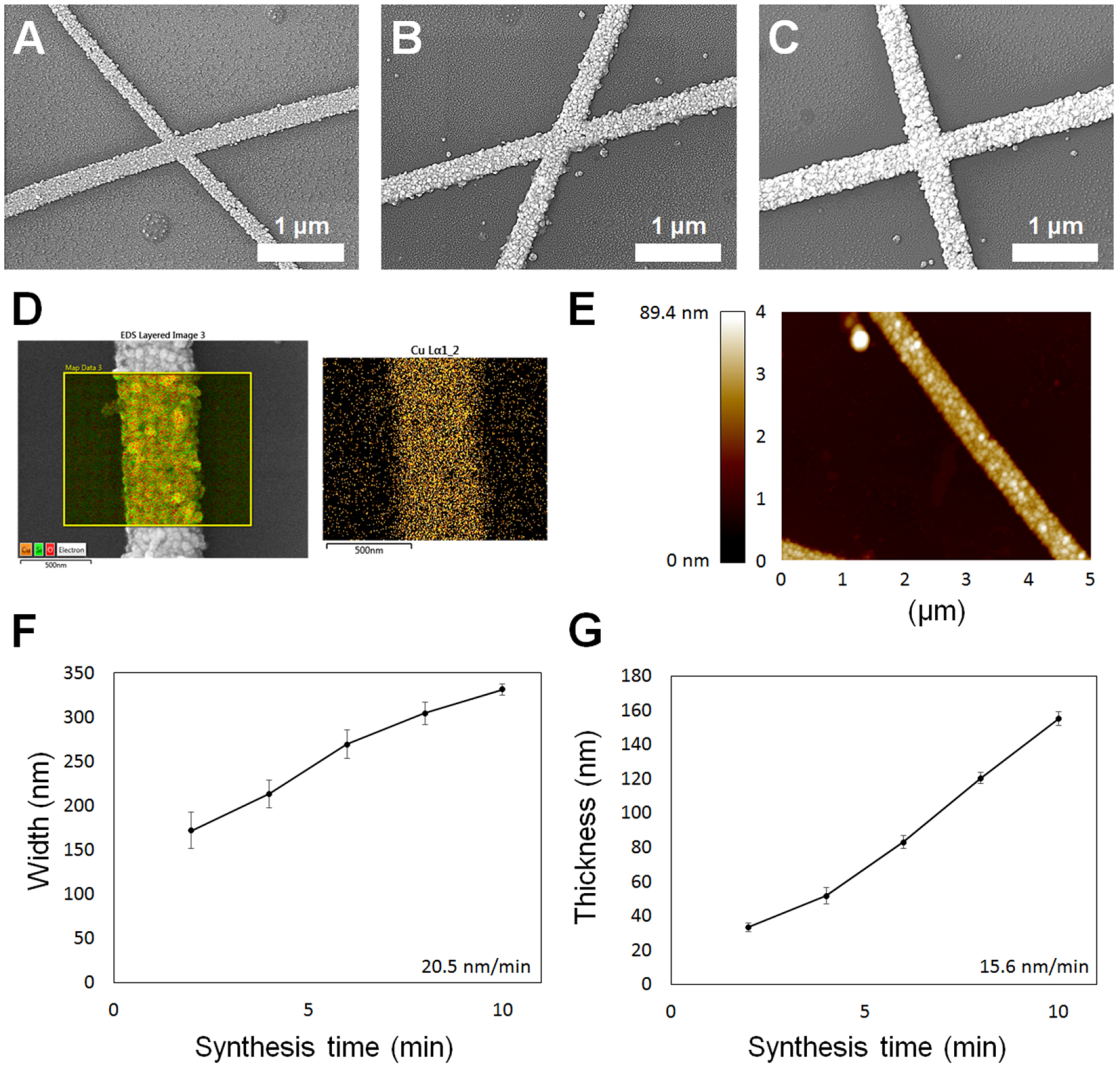
Analysis of copper nanowire fabrication conditions and morphology. (**A**–**C**) Scanning electron microscopy (SEM) images of copper nanowires deposited for 2, 6, and 10 min, respectively. (**D**) Fabricated copper nanowire and its component analysis maps determined by energy dispersive X-ray spectroscopy (EDS). (**E**) Atomic force microscopy (AFM) analysis map showing nanowire thickness and morphology. (**F**) Graph showing correlation between electroless deposition time and nanowire width (n = 5, mean ± standard error). (**G**) Graph showing correlation between electroless deposition time and nanowire thickness (n = 5, mean ± standard error).

Figure 2 shows the change in morphology of the nanowire under copper electroless deposition. Figure 2A–C shows electron microscope observations of electroless deposition at 2, 6, and 10 min, respectively. The overlap between two nanofibers that could cause junction resistance was observed along with a 2-D morphology; there was no difference in height between conducting nanofibers, unlike the studies of transparent electrodes fabricated using conventional electrospinning and electroless deposition^19^. This is because the heat treatment facilitates formation of a single seed layer on which electroless deposition is conducted. EDS component analysis was conducted to confirm that the copper electroless deposition was successful, and the copper component was detected along the nanowire, as shown in Fig. 2D. Figure 2E shows AFM analysis results of a nanowire synthesized with peak positions of 89.4 nm. Scanning electron microscopy (SEM) and AFM images showed that the shape of the nanofiber was completely flat without defects at the junction; also, the 2-D shape differed from those of cylindrical nanofibers fabricated in previous studies. Based on these SEM images, the width of the copper nanowire with respect to the synthesis time was analyzed (Fig. 2 F). The synthesis time and width were proportional, and the width grew at an average rate of 20.5 nm/min. The analysis results of nanowire thickness with respect to synthesis time from AFM measurements is shown in Fig. 2G; the thickness was proportional to the synthesis time, and the average growth rate was 15.6 nm/min. These results indicate that the nanofiber dimensions and morphology can be successfully controlled through the solution process, and that the fabricated nanofiber network is a 2-D structure with no junction resistance. The ability to actively control the density and morphology of nanofibers allows the sheet resistance to be tailored to the application while minimizing the reduction in transmittance.

**Figure 3:**
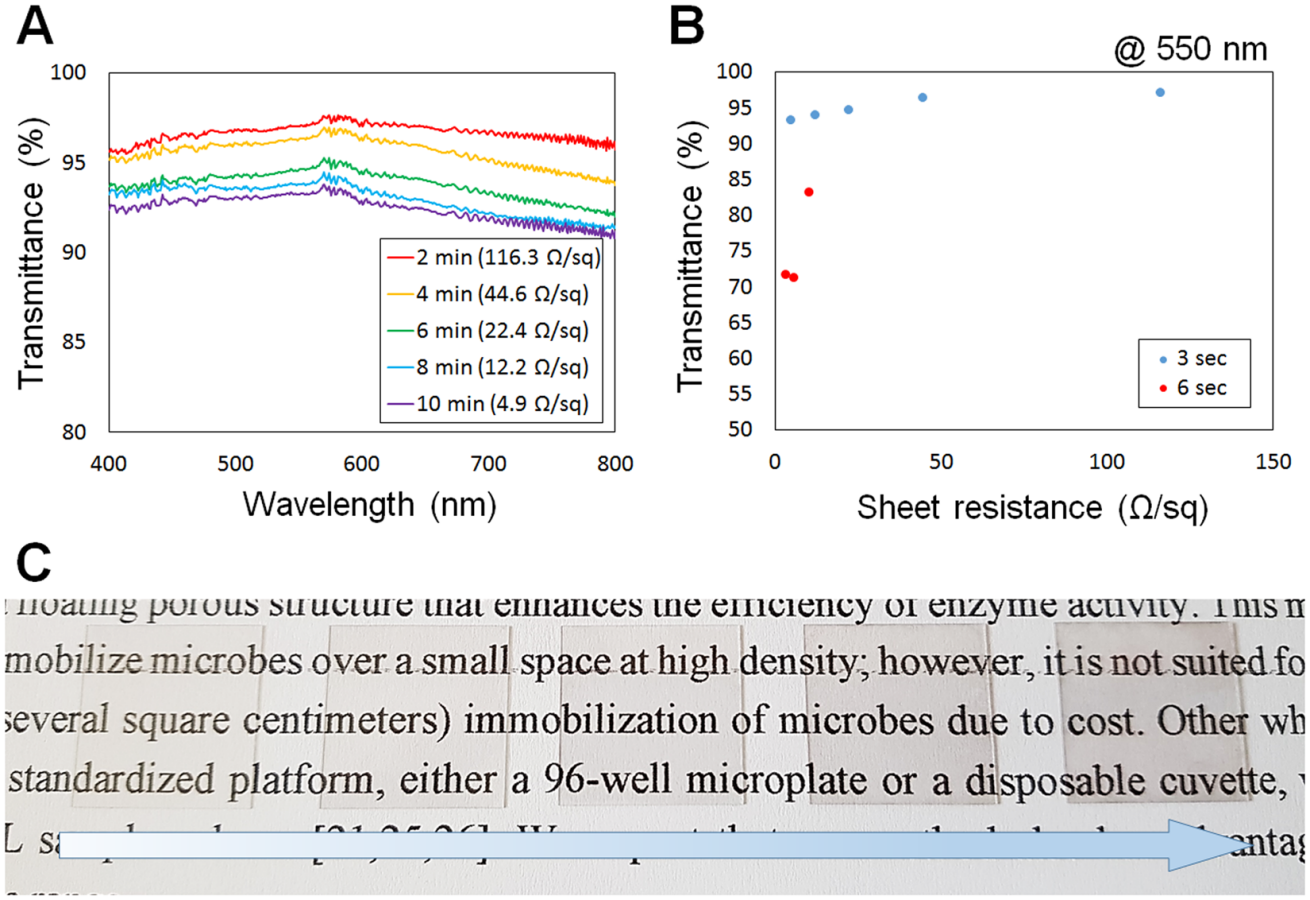
Correlation between sheet resistance after electroless deposition and conductivity of fabricated transparent electrodes. (**A**) Graph of the correlation between transmittance and sheet resistance (in the visible range: 400–800 nm). (**B**) Graph showing the correlation between sheet resistance and transmittance at a wavelength of 550 nm. The blue dot and the red dot indicate the nanofiber collection time adjusted to 3 s and 6 s, respectively. (C) Transparent electrodes fabricated using deposition times of 2, 4, 6, 8, and 10 min, respectively.

Figure 3 shows the relationship between transmittance and sheet resistance of the fabricated transparent electrode. Figure 3A shows the transmittance of the electrode over the wavelength range of 400–800 nm with respect to the sheet resistance. The lower the sheet resistance, the lower the transmittance. It was confirmed that even if the synthesis was performed for a long time to obtain a low sheet resistance (4.9 Ω/sq), a transmittance of 90% or more was observed across the entire visible range. In addition, by varying the electroless deposition time of samples with the same nanofiber density, the sheet resistance varied from several Ω/sq to several hundred Ω/sq while maintaining a transmittance of over 90%. Figure 3B shows the correlation between sheet resistance and transmittance at 550 nm. Controlling the collection time for the electrospun nanofibers allows control over the seed layer density (3 s, blue dots). Even if the collection time is doubled (6 s, red dots), the sheet resistance did not decrease significantly; however, the transmittance dropped off sharply. Figure 3C shows the transparent electrodes fabricated using deposition times of 2, 4, 6, 8, and 10 min, in order, placed on the top of a sheet of paper. Even the device with the longest electroless deposition time was almost transparent to the naked eye.

**Figure 4:**
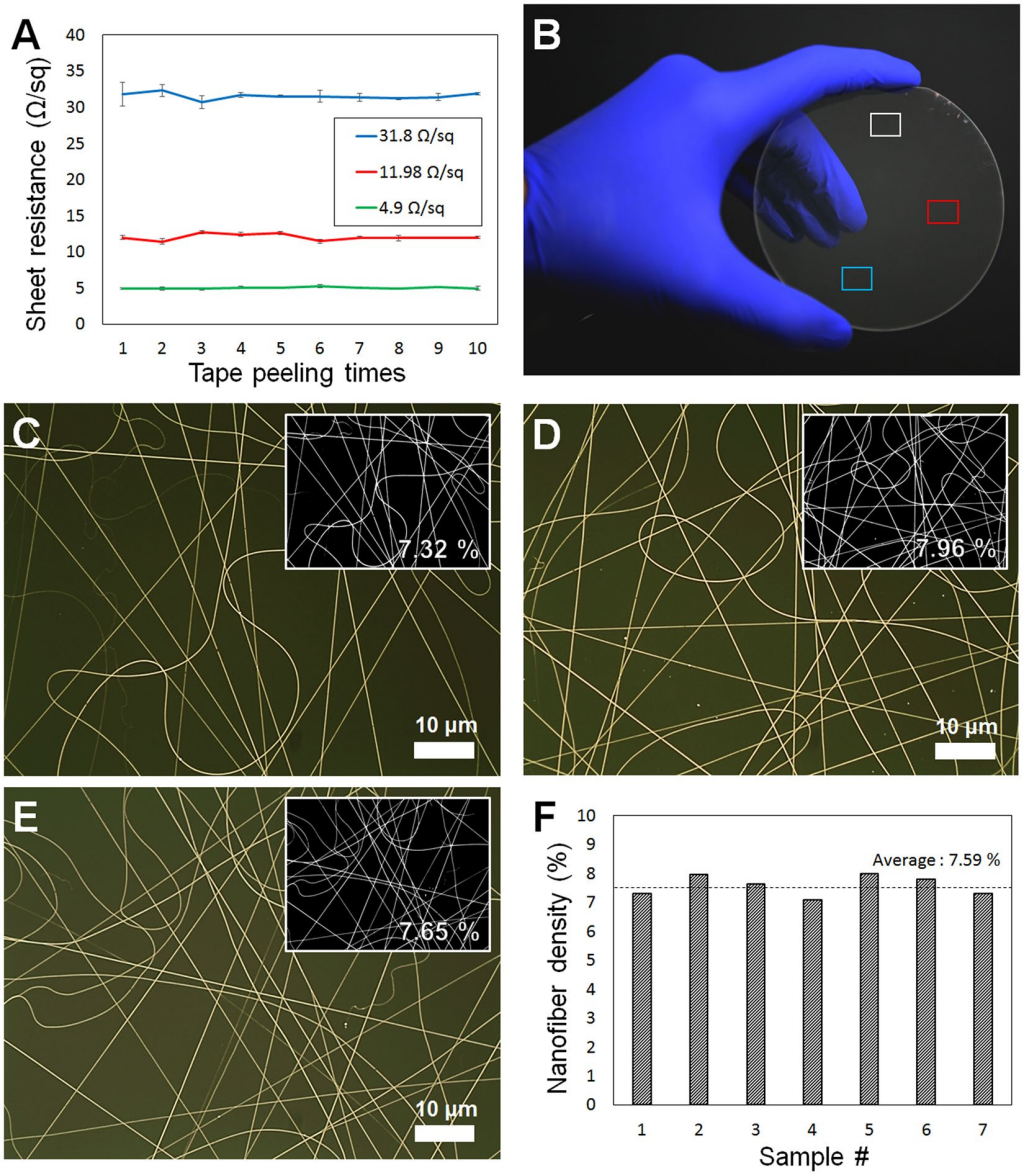
Mechanical stability test results of transparent electrodes and electrodes evenly formed on a 4-inch glass wafer. (**A**) Commercial 3 M Scotch Tape was placed in direct contact with the electrode, repeatedly removed and reapplied, and the change in sheet resistance measured (n = 5, mean ± standard error). The electrode formed evenly over the 4-inch glass wafer (**B**); each part was observed with an optical microscope (**C**–**E**). The small inset numbers and pictures for (**C**–**E)** are the ratio of the area occupied by the nanowire in the total area and the black/white image. (**F**) A graph showing the nanowire density calculations for seven randomly selected areas in 4-inch glass wafer.

In previous studies, electrodes made using electrospinning were found to have limited mechanical strength, because cylindrical electrodes were placed on a target substrate. In this study, however, superior adhesion between the substrate and the metal electrode was observed, because the electrode was produced via electroless deposition on a seed layer. To demonstrate this, repeated tape peeling was performed using commercially available 3 M Scotch Tape, and the change in sheet resistance was measured (Fig. 4A). The sheet resistance did not change after ten cycles of Scotch Tape adhesion to the metal nanostructure. In addition to mechanical durability, this direct electrospinning method can be more easily achieved over a large area compared with conventional transfer processes that require complex fabrication conditions. Electrodes were also fabricated on a 4-inch glass wafer, to demonstrate deposition of the metal electrodes over a large area using the motorized stage (Fig. 4B). A metal electrode formed uniformly over the entire area and could be seen with the naked eye. Optical microscope images of the other parts of the wafer showed that the metal electrode formed uniformly (Fig. 4C–E). The black / white images shown in inset in C, D and E are the images in which the part with nanowire is white and the part without nanowire is black with image J program. Using this to calculate the area of the white area in the whole area and the results are small numbers in the inset images. (C: 7.32%, D: 7.96%, E: 7.65%) In this way, 7 regions are randomly selected in the glass wafer and the nanowire density of each part is calculated. The result is shown in Fig. 4F. As a result, the average nanowire density was 7.59% and the standard error was 0.32. Therefore, the transparent electrode fabrication technique proposed in this study is a very uniform and reliable method. These results demonstrate the potential for large-area, mass production with guaranteed mechanical strength for practical industrial applications.

**Figure 5:**
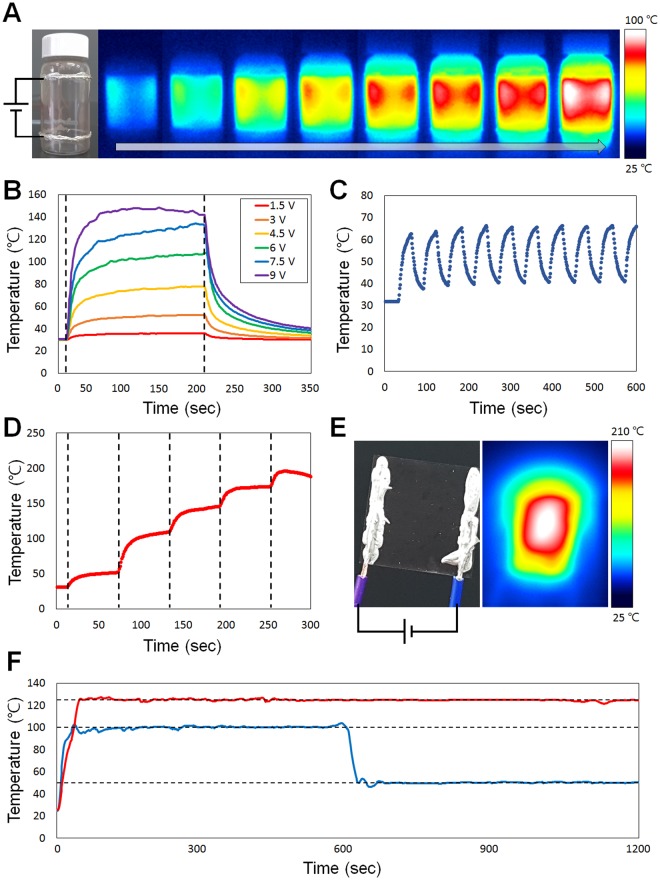
Performance evaluation of transparent electrode used as a heater. (**A**) A copper nanowire was fabricated on the outside of the commercial vial, a current was passed, and the heating pattern was photographed with an infrared camera over time. (**B**) Various voltages were applied to the electrodes and the temperature changes observed. (**C**) On/off voltage and observation of temperature change. (**D**) Measuring the maximum temperature of the electrode, which was stepped up gradually to a higher voltage. (**E**) Observation of the electrode with a thermal imaging camera at the maximum temperature. (**F**) A graph showing thermal stability of fabricated transparent heater. The red curve shows that the temperature is steadily maintained at 120 degrees Celsius and the blue curve shows that the temperature is kept at 100 degrees Celsius and then kept at 50 degrees Celsius and steady. The English in this document has been checked by at least two professional editors, both native speakers of English. For a certificate, please see: http://www.textcheck.com/certificate/LkcBh1

The performance of the transparent electrode was verified by measurements taken while using the electrode as a heater. Figure 5 shows time-dependent temperature profiles measured when a voltage was applied to the fabricated transparent heater. Figure 5A shows the results of forming an electrode on the surface of a commercial vial and passing a current through the electrode to form a heater on a curved surface. To collect the electrospun nanofiber on the curved surface (20 mL glass vial, radius of curvature: 14 mm), the target substrate was placed on the conductor collector and the desired surface was evenly exposed to the syringe where the nanofiber was ejected. A uniform high-temperature region formed between the two electrodes, after a voltage of 5 V was applied, and the temperature of the entire vial increased. Uniform electrode formation over the curved surface was confirmed, as well as successful operation as a heater. Figure 5B–E show measurements of the temperature change when an electrode was formed on a 1.8 cm × 1.8 cm glass slide; a thermocouple was connected to the back of the glass. Figure 5B shows the measured maximum temperature with applied voltages. Voltages from 1.5 V to 9 V at intervals of 1.5 V were used, and the corresponding currents were 0.04, 0.09, 0.14, 0.18, 0.20, and 0.25 A, respectively. A higher applied voltage led to a higher maximum temperature, because the power was applied to the heater electrode as Joule heat. Figure 5C shows the measured temperature results for a voltage (6 V) turned on and off repeatedly at 30-s intervals. It was confirmed that the heating performance of the heater stayed constant even after 10 repeat cycles. Figure 5D shows the temperature change as the voltage was increased every minute to measure the maximum temperature at which the electrode could be used as a heater. The dotted lines on the graph represent voltages of 5, 10, 12.5, 15, 17.5, and 20 V. When the voltage exceeded 20 V, too much heat was generated and the electrode was destroyed, leading to a drop in temperature. Because the thermocouple measures the temperature at the back of the glass, it does not reflect the real-time temperature of the electrode. A thermal image of the electrode at the highest temperature is shown in Fig. 5E, indicating a maximum localized temperature of 210 °C. Figure 5F shows thermal stability of fabricated transparent heater. The blue line shows a result of maintaining a temperature of 100 °C for the first 10 minutes and a temperature of 50 °C for 10 minutes after. Also, the red graph is the result of maintaining 125 °C for 20 minutes. The target temperature was maintained well without changing even at high temperature. These results confirm that the proposed transparent heater fabrication method is capable of direct fabrication on a 3-D complex surface without transferring the conducting nanofiber web, and that the performance of the heater is also robust to practical applications.

## Conclusion

In conclusion, we developed an electrospinning technique to overcome the limitations of transparent electrode fabrication. A random fiber network was fabricated on the substrate using electrospinning and heat treated to form a thin seed layer. The fabricated seed layer was used to form an electrode via electroless deposition, a solution process; the sheet resistance and transparency were controlled by controlling the reaction time. This technology can be used to form electrodes on complex surfaces, similar to other electrospinning-based transparent electrode fabrication techniques, with the advantages of being polymer-free and having zero junction resistance due to the heat treatment process. In addition, the adhesion at the interface between the substrate and the electrode part where the transparent electrode forms is relatively strong, and the electrode is formed using a bottom-up solution process wherein the characteristics of the electrode can be actively controlled by controlling the reaction time. Given that the density of the electrospun nanofiber can be adjusted by controlling the collection time, the method can be widely applied to electrostatic touch panels, touch screens, LEDs, and solar cells.

## Electronic supplementary material

supplementary materials
