# Supplementary material for: Junction-free Flat Copper Nanofiber Network-based Transparent Heater with High Transparency, High Conductivity, and High Temperature
Source: Sci Rep. 2018 Sep 11;8:13581. doi: 10.1038/s41598-018-32045-6 (PMC6134026; doi:10.1038/s41598-018-32045-6)
Supplement: Supplementary file 1 — supplementary materials [file 41598_2018_32045_MOESM1_ESM.doc]

Junction-free Flat Copper Nanofiber Network-based Transparent Heater with High Transparency, High Conductivity, and High Temperature

*Geon Hwee Kim ^1^, Jung Hwal Shin ^2^, Taechang An ^3, *^ and Geunbae Lim ^1, *^*

1. Department of Mechanical Engineering, Pohang University of Science and Technology (POSTECH), Pohang 790-784, Republic of Korea
2. Biomedical Engineering, School of Life Sciences, Ulsan National Institute of Science and Technology (UNIST), 50, UNIST-gil, Ulsan 44919, South Korea
3. Department of Mechanical Design Engineering, Andong National University, Kyungbuk, 760-749, Republic of Korea

* Corresponding author: Geunbae Lim (limmems@postech.ac.kr) and Taechang An ([tcmerias@anu.ac.kr](mailto:tcmerias@anu.ac.kr))

KEYWORDS: Transparent electrode, copper, electroless deposition, electrospinning, junction resistance


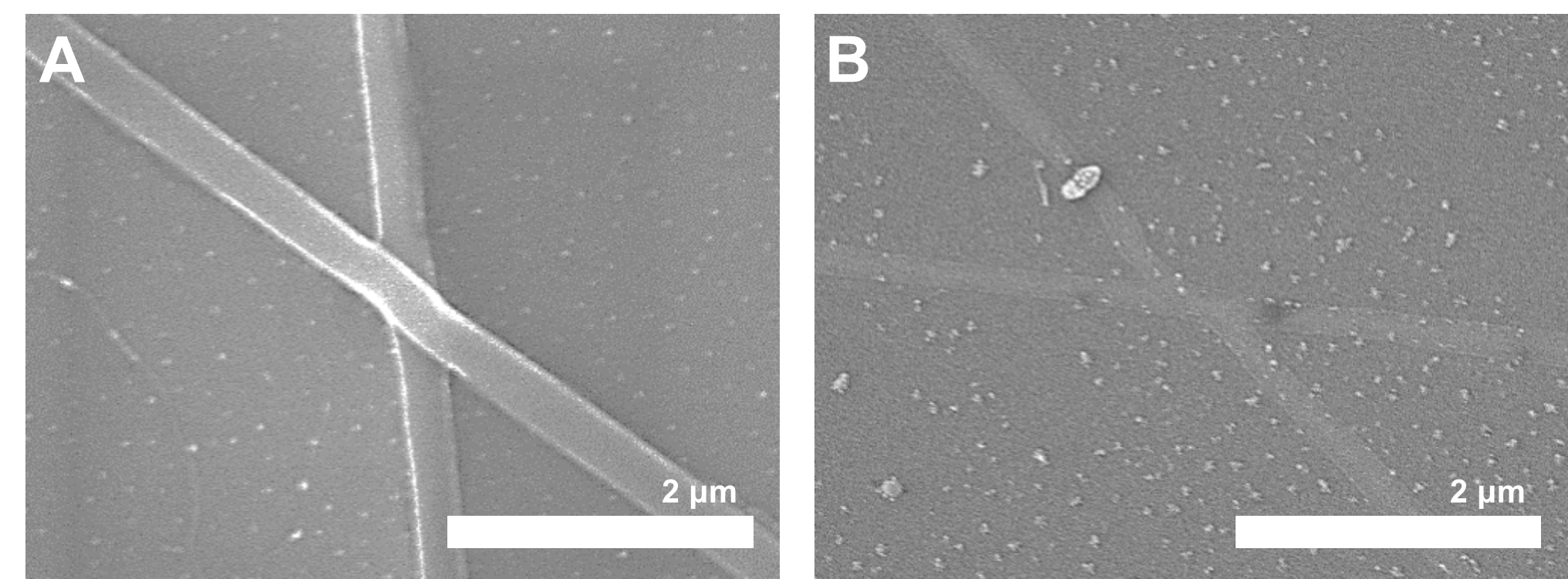


Figure S1. Difference in shape of electrospun nanofiber before and after heat treatment. (A) Electrospun nanofibers before heat treatment. (B) Electrospun nanofibers after heat treatment (seed layer)


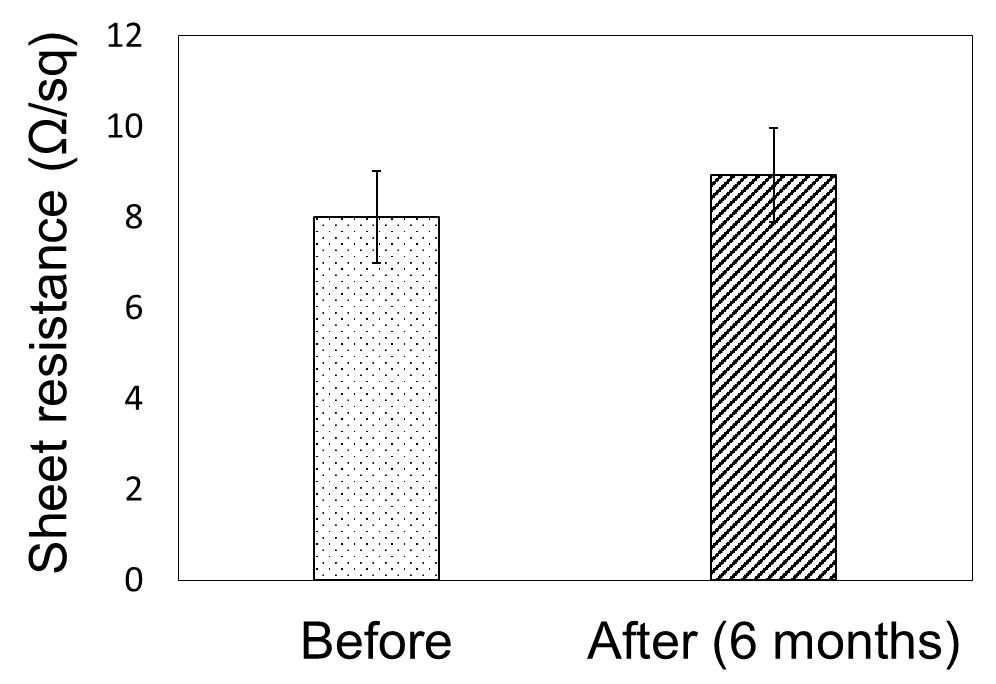


Figure S2. Graph showing changes in sheet resistance of transparent electrodes after 6 months of fabrication (n = 5, mean ± standard error).
